# Treatment of cholecysto-choledocolithiasis in elderly patients: personal experience

**DOI:** 10.1186/1471-2318-11-S1-A53

**Published:** 2011-08-24

**Authors:** Agostino Ricotta, Maria Sofia, Saverio Latteri, Francesca R Cannemi, Gaetano La Greca, Domenico Russello

**Affiliations:** 1 Department of Emergency Surgery, Cannizzaro Hospital, Catania, Italy

## Background

Biliary stones are a common disease in western countries, and its management has changed dramatically over the past decade. Due to the increase of the middle-aged people, a larger number of elderly patients are affected by this disease. Laparoscopic cholecystectomy (LC) represents the gold standard for the treatment of gallbladder stones, and in cholecystectomized patients, the treatment of CBD stones remains the exclusive work of the endoscopist. But if the patient still has the gallbladder with stones, the ideal management of CBD stones remains controversial. There are two treatment options: the two time (LC and pre or postoperative ERCP), and the one time procedures (trans cystic approach and CBD exploration and laparoendoscopic procedure called “rendezvous”:RV). The aim of the work is to analyse our results of the mininvasive treatment of the cholecysto-choledocolithiasis in elderly patients.

## Materials and methods

In the period between September 2008 and November 2010, all patients affected by CBD stones and admitted to the Department of Emergency Surgery of Cannizzaro Hospital in Catania, were analyzed, and from this group patients aged >65 years were considered for the present study. Age, sex, main clinical data, history, diagnosis, type of treatment, postoperative complications, length of hospital stay and mortality were recorded. Patients affected by cholecysto-choledocolithiasis were submitted to the LC and RV technique. If the patients couldn’t be submitted to this procedure because of high anaesthetic risks, ERCP was performed. However ERCP was always performed in cholecistectomized patients with jaundice, biliary pancreatitis, cholangitis and imaging showing CBD stones.

## Results

In the period of the study we observed 68 patients with CBD stones, 37 were older than 65 years: 15 (40.5%) males and 22 (59.5%) female, with a mean age of 76.45 years (range 65 -93). Twelve (32.4%) patients were treated by LC and intraoperative clearance of the CBD by the RV. ERCP was performed in 22 (59.5%) patients: 8(21.6%) previous cholecistectomized and 14(63.6%) who still had their gallbladder, but with high anaesthetic risk. In one patient the RV technique failed and laparoscopic choledocotomy was performed and a T-tube left in situ, but after 3 week trans-Kher cholangiography showed residual stones, so the patient was submitted to ERCP. In other two cases only LC was performed because the papilla of Vater was difficult to approach. The length of hospital stay was on average 7 days. Postoperative complications occurred in 7(20%) patients: one patient developed post-ERCP pancreatitis, two patients post-ERCP increasing of sieric amylase and lipase, three patients with early stones recurrence, one patient developed post- ERCP cholecystitis. Only one death was recorded.

## Conclusions

For the treatment of the cholecysto-choledoco lithiasis, the RV technique is the best option, even in the elderly, because the morbidity and the risk of iatrogenic damage seem lower than ERCP. However in an older high risk patient the ERCP remains a good therapeutical option, achieving an acceptable risk of postoperative complications.

